# Supplementary material for: The adapt-to-nutrient NRPS-like secondary metabolite gene cluster facilitates Verticillium dahliae adaptation to different nutrient environments
Source: PLoS Genet. 2026 Mar 31;22(3):e1011930. doi: 10.1371/journal.pgen.1011930 (PMC13065033; doi:10.1371/journal.pgen.1011930)
Supplement: S1 Fig — (DOCX) [file pgen.1011930.s001.docx]

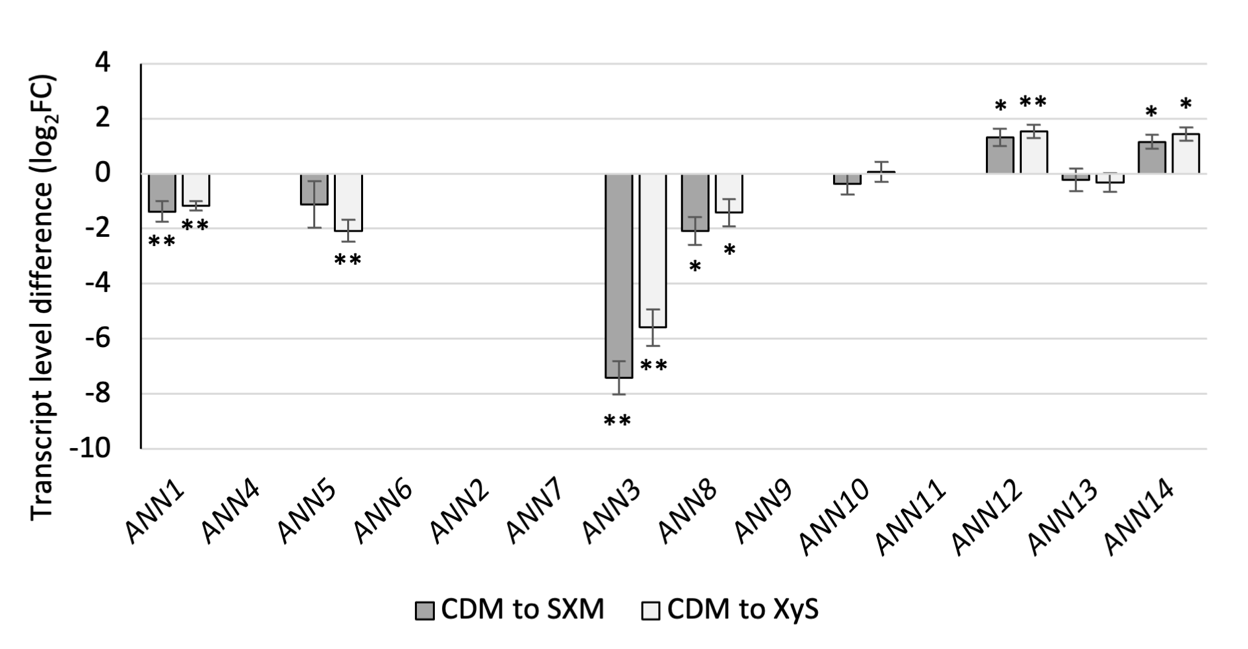


**S1 Fig. *ANN* cluster genes are expressed similarly in tomato xylem sap as in the pectin-rich SXM.** Transcript level of the core biosynthetic gene *ANN3* is 171-fold lower in SXM and 48-fold lower in tomato xylem sap (XyS) in comparison to the transcript level in minimal medium (CDM). Expression of the transcription factor-encoding *ANN1* is more than two-fold lower in SXM and XyS compared to CDM. Expression levels of the transport-related gene *ANN5* and the gene of unknown function *ANN8* are down-regulated in SXM and XyS, whereas the biosynthetic gene *ANN14* and the transport-related gene *ANN12* are up-regulated. The difference in transcript levels is log_2_-transformmed and plotted on the y-axis. Unpaired t-test was performed to compare the counts of transcripts of each *ANN* cluster genes in the respective culturing condition (*, P < 0.05; **, P < 0.01).
