# Supplementary material for: The adapt-to-nutrient NRPS-like secondary metabolite gene cluster facilitates Verticillium dahliae adaptation to different nutrient environments
Source: PLoS Genet. 2026 Mar 31;22(3):e1011930. doi: 10.1371/journal.pgen.1011930 (PMC13065033; doi:10.1371/journal.pgen.1011930)
Supplement: S2 Fig — (DOCX) [file pgen.1011930.s002.docx]

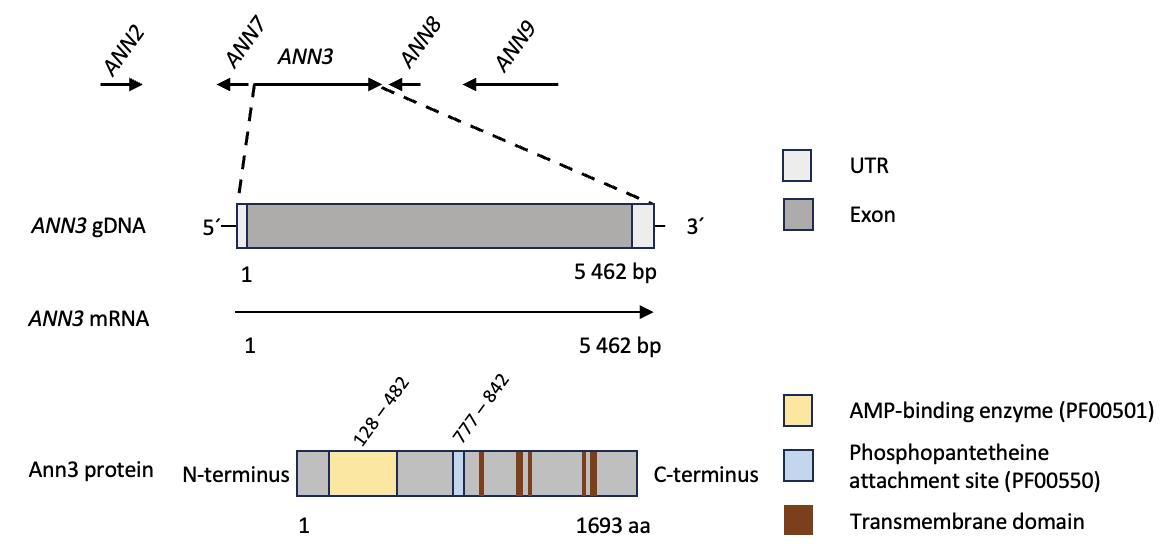


**S2 Fig. The genomic structure of the core biosynthetic enzyme-encoding gene *ANN3*.** The 5462 bp *ANN3* gene contains 1 exon (dark grey), and 5’- and 3’ UTRs (light grey). The 1693 aa Ann3 protein contains an AMP-binding enzyme domain (PF00501; 128 – 482 aa; yellow), a Phosphopantetheine attachment site (PF00550; 777 – 842 aa; light blue), and five Transmembrane domains (919 – 940, 1102 – 1129, 1154 – 1177, 1418 – 1439, 1468 – 1496; brown).
