# Supplementary material for: The adapt-to-nutrient NRPS-like secondary metabolite gene cluster facilitates Verticillium dahliae adaptation to different nutrient environments
Source: PLoS Genet. 2026 Mar 31;22(3):e1011930. doi: 10.1371/journal.pgen.1011930 (PMC13065033; doi:10.1371/journal.pgen.1011930)
Supplement: S3 Fig — (DOCX) [file pgen.1011930.s003.docx]

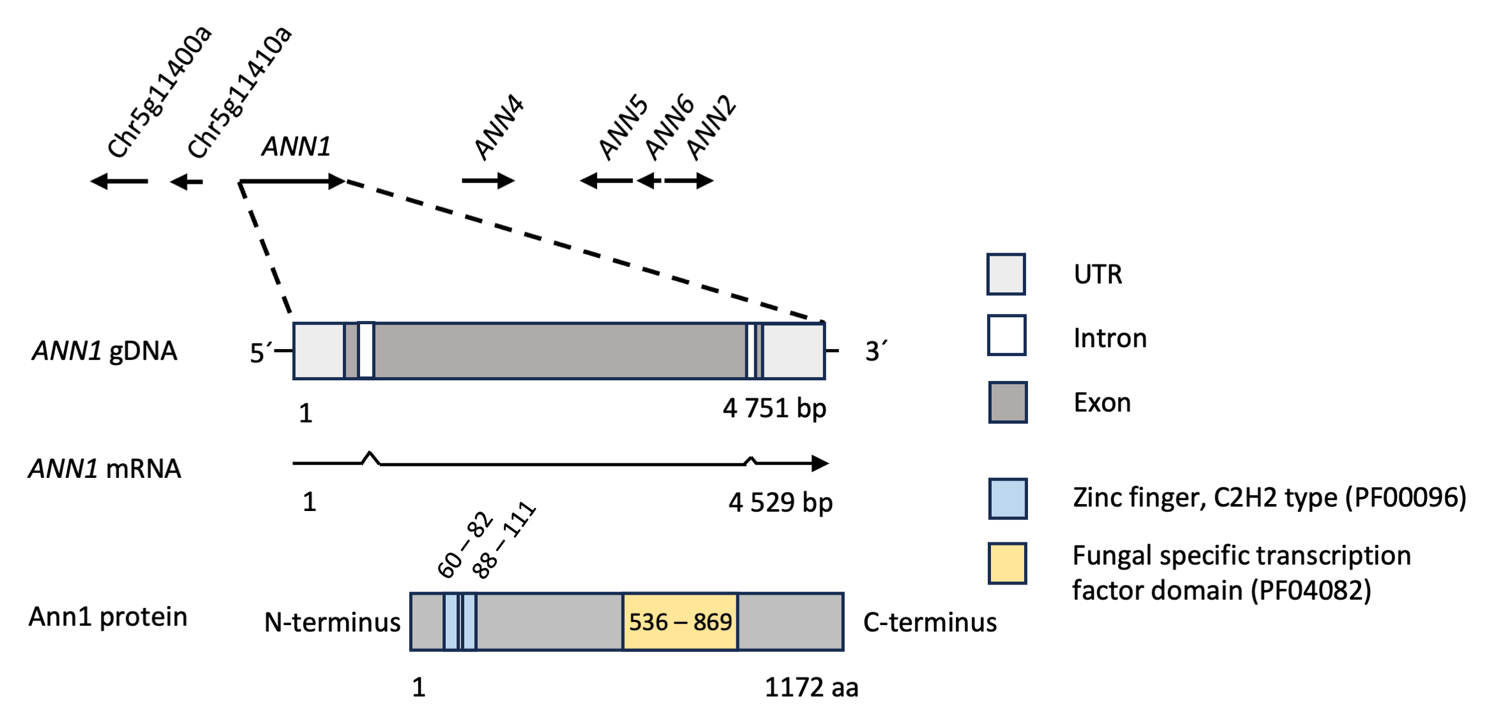


**S3 Fig. The genomic structure of the transcription factor-encoding gene *ANN1*.** The 4751 bp regulatory gene *ANN1* contains 2 introns (white), 2 exons (dark grey), and 5’- and 3’ UTRs (light grey). The 1177 aa Ann1 protein contains a C2H2-type zinc finger domain (PF00096; 60 – 111 aa; light blue) and a fungal specific transcription factor domain (PF04082; 536 – 869, yellow).
