# Supplementary material for: The adapt-to-nutrient NRPS-like secondary metabolite gene cluster facilitates Verticillium dahliae adaptation to different nutrient environments
Source: PLoS Genet. 2026 Mar 31;22(3):e1011930. doi: 10.1371/journal.pgen.1011930 (PMC13065033; doi:10.1371/journal.pgen.1011930)
Supplement: S7 Fig — (DOCX) [file pgen.1011930.s007.docx]

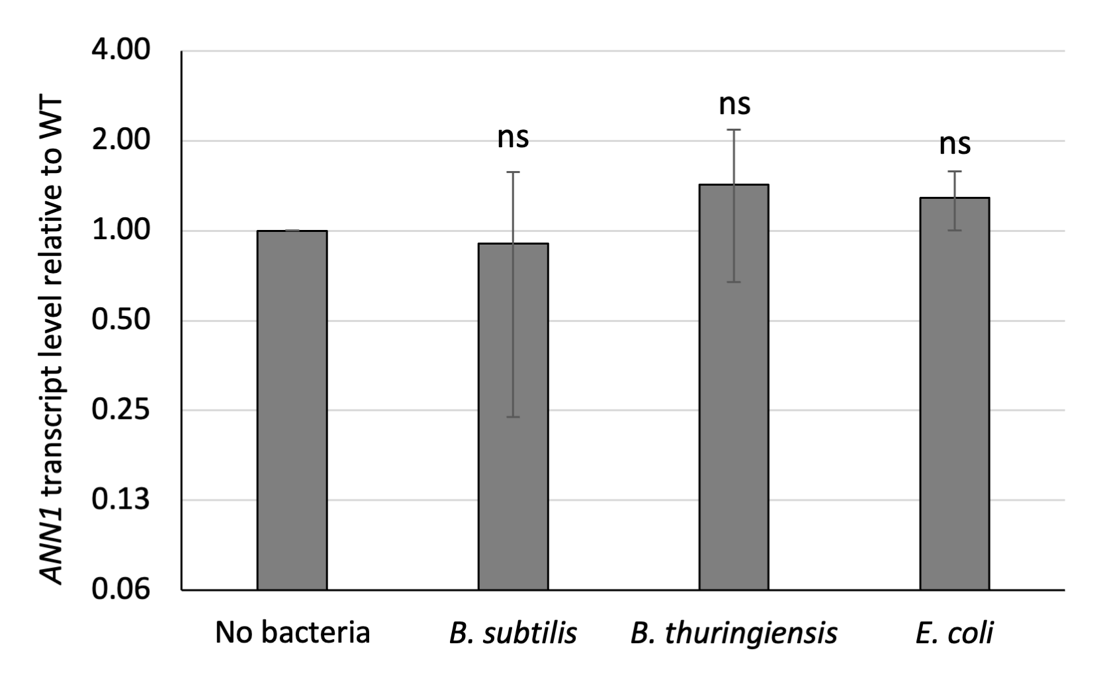


**S7 Fig. Co-cultivation of *V. dahliae* with bacteria does not affect *ANN1* transcript levels.** Transcript levels of *ANN1* in the *V. dahliae* WT pure culture (no bacteria) and *V. dahliae* WT co-cultured with *B. subtilis* 168, *B. thuringiensis* GOE4, or *E. coli* DH5⍺ for 24 hr were analysed by qRT-PCR. Compared to the pure culture expression levels, transcript levels of *ANN1* showed no significant difference in any co-cultured samples. The y-axis is plotted on a log_2_ scale. All samples were cultured in liquid PDM for 5 days before the mycelia were harvested for RNA extraction. Bacterial cultures were inoculated to the co-cultured samples 4 dpi at an initial OD_600_ of 0.01. One-sample t-test was performed to compare transcript levels of each tested condition to the WT expression levels (ns, not significantly different).
