# Supplementary material for: The adapt-to-nutrient NRPS-like secondary metabolite gene cluster facilitates Verticillium dahliae adaptation to different nutrient environments
Source: PLoS Genet. 2026 Mar 31;22(3):e1011930. doi: 10.1371/journal.pgen.1011930 (PMC13065033; doi:10.1371/journal.pgen.1011930)
Supplement: S10 Fig — (DOCX) [file pgen.1011930.s010.docx]

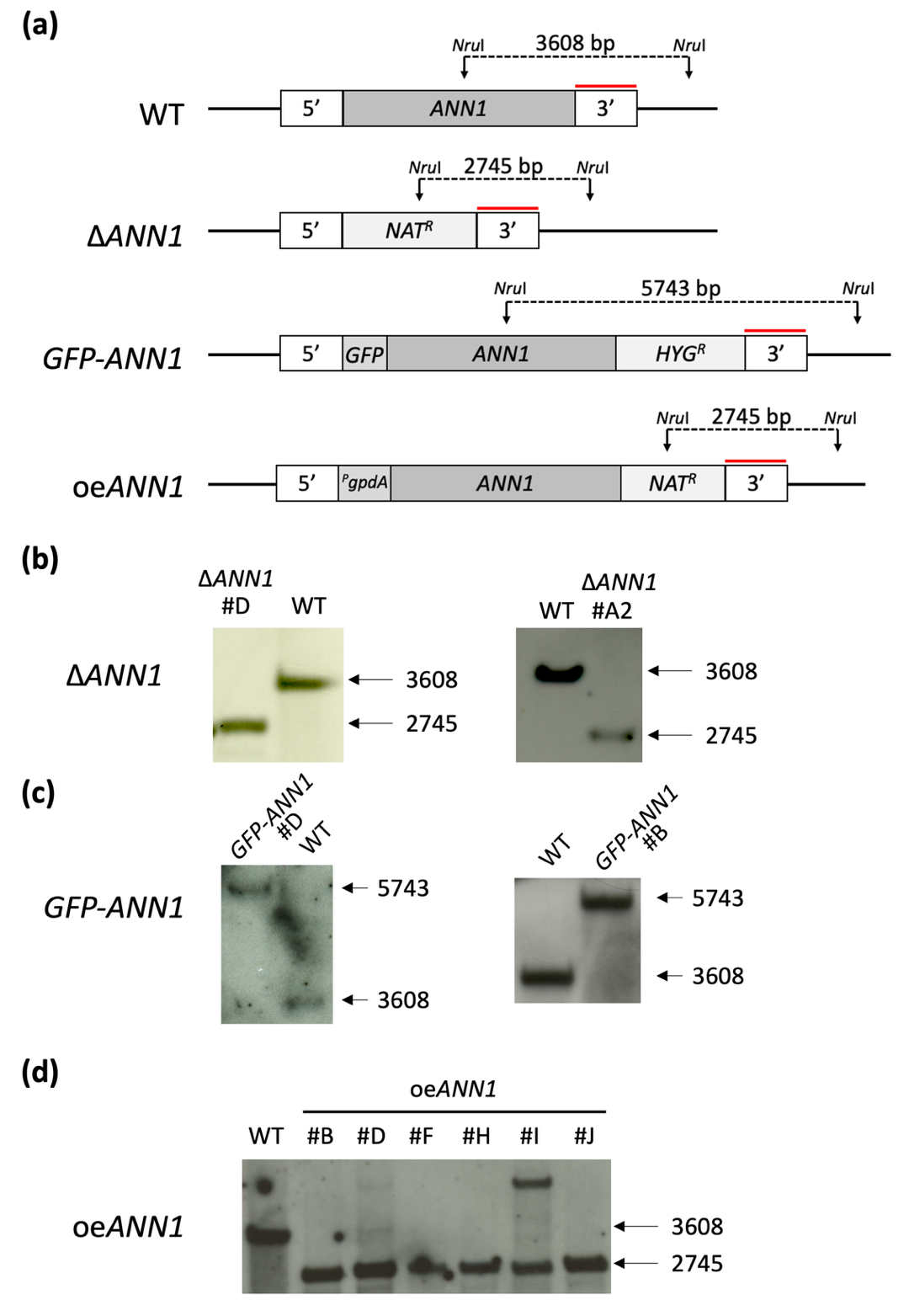


**S10 Fig**. **Verification of the *V. dahliae ANN1* mutant strains.** (a) Schemes of the genome of the WT and *ANN1* mutant strains and the mutant strains are depicted. *Nru*I ristriction sites are labelled in black arrows, and the probe that binds to the 3’ flanking region of *ANN1* are labelled in red line. The expected fragment sizes are written in the scheme. (b) The genome of ∆*ANN1* isolates D and A2 were confirmed to be correct. The WT strain served as control. (c) The genome of *GFP-ANN1* isolates D and B were confirmed. The WT served as control. (d) The genome of oe*ANN2* isolates H and J were confirmed. Isolates D and I were incorrect, and isolates B, D, F, and I were not used for further studies. The WT strain served as control.
